# Microfluidic transfection of mRNA into human primary lymphocytes and hematopoietic stem and progenitor cells using ultra-fast physical deformations

**DOI:** 10.1038/s41598-021-00893-4

**Published:** 2021-11-01

**Authors:** Jocelyn Loo, Ian Sicher, Ailin Goff, Ockchul Kim, Nicole Clary, Alexander Alexeev, Todd Sulchek, Alla Zamarayeva, Sewoon Han, Miguel Calero-Garcia

**Affiliations:** CellFE, Inc., Suite 110, 980 Atlantic Ave, Alameda, CA 94501 USA

**Keywords:** Transfection, Molecular medicine, Biomedical engineering, Haematopoietic stem cells, Permeation and transport

## Abstract

Messenger RNA (mRNA) delivery provides gene therapy with the potential to achieve transient therapeutic efficacy without risk of insertional mutagenesis. Amongst other applications, mRNA can be employed as a platform to deliver gene editing molecules, to achieve protein expression as an alternative to enzyme replacement therapies, and to express chimeric antigen receptors (CARs) on immune cells for the treatment of cancer. We designed a novel microfluidic device that allows for efficient mRNA delivery via volume exchange for convective transfection (VECT). In the device, cells flow through a ridged channel that enforces a series of ultra-fast and large intensity deformations able to transiently open pores and induce convective transport of mRNA into the cell. Here, we describe efficient delivery of mRNA into T cells, natural killer (NK) cells and hematopoietic stem and progenitor cells (HSPCs), three human primary cell types widely used for ex vivo gene therapy applications. Results demonstrate that the device can operate at a wide range of cell and payload concentrations and that ultra-fast compressions do not have a negative impact on T cell function, making this a novel and competitive platform for the development of ex vivo mRNA-based gene therapies and other cell products engineered with mRNA.

## Introduction

The use of mRNA as a vehicle for therapeutic application has become more practical with the advancement of novel nonviral delivery methods^1,2^. Amongst other advantages, mRNA offers drug developers an efficient, easy-to-design, and safer platform for gene transfer compared to DNA transfection or viral vector transduction^3^. Unlike DNA-based therapies, mRNA becomes functional once it reaches the cytoplasm, resulting in transient expression of a therapeutic protein. In this way, mRNA carries a reduced risk of genotoxicity because it is unable to integrate into the host genome^4,5^. Moreover, transient expression is a feature that can be exploited when addressing safety concerns associated with long-term expression of an experimental transgene^4^. Pharmaceutical companies such as Sanofi, Sangamo, Pfizer, Myeloid TX, Carisma Therapeutics, Moderna, and AstraZeneca have invested money to advance their presence in the mRNA therapeutics space^6–9^. Clinical applications of mRNA span across multiple indications, encompassing both in vivo and ex vivo approaches. In vivo mRNA delivery has been employed for interventions such as protein replacement therapies, cell reprogramming and gene editing, amongst others^10^. However, it is probably in vivo mRNA vaccination that has most recently and noticeably underscored the safety advantages of mRNA-based interventions^10^. In December 2020, Pfizer-BioNTech COVID-19 Vaccine became the fastest vaccine to receive Emergency Use Authorization by the FDA ever, while also being the first mRNA vaccine to receive said approval by regulators^11^.

In the ex vivo space, mRNA transfection is notably being developed as an alternative modality for cancer immunotherapy, in juxtaposition to integrating viral vectors^12^. Chimeric antigen receptors (CARs) are engineered proteins that repurpose cellular receptor domains together with antibody-based recognition domains, in order to retarget immune cells (e.g., T and NK cells) against antigens of interest, such as tumor antigens. T cells made with mRNA-encoded CARs offer a time and cost-effective method against their retroviral vector CAR counterparts^3^. Integrating viral vectors carry a risk of insertional mutagenesis and potential safety issues that stem from long-term persistence of engineered T cells, including immune-mediated toxicity from cytokine storm, cardiac arrhythmia, or respiratory failure^13^. The transient nature of mRNA enables dose-escalation throughout treatment without the risk of permanent T cell activity, as RNA degradation ensures the removal of CARs in patients without needing suicide or switch induction systems^3,13^. In addition to CAR T, CAR NK cells have also been rapidly gaining interest in the cancer immunotherapy field. CAR NK cells have demonstrated several advantages over CAR-T in regard to safety, alloreactivity, and pathways to eliminate tumor cells^14–16^. Transient expression with mRNA can also be harnessed for permanent genomic modification by means of delivering gene editing molecules. Zinc Finger nuclease (ZFN), transcription activator-like effector nucleases (TALEN), and Cas-9 mRNA have all been used to edit human T cells, and hematopoietic stem cells amongst other cell types^17^. The combination of transient and permanent gene modifications that ex vivo mRNA gene therapy provides strengthen its position in the future of the field provided an efficient mode of manufacture of cell products develops.

There are currently several methods for ex vivo mRNA transfection in human primary hematopoietic cells (e.g. lymphocytes), with the clinical standard being electroporation. Short high voltage electrical pulses are used to generate transient pores on the cell membrane allowing transfection. Irreversible electroporation has proven to be an issue for electroporation as perpetual permeabilization of the cell membrane caused by electrical pulse can lead to substantial cell death and compromises to cell viability^18^. The usage of electric fields to enable transfection causes high toxicity and low cell viability; with cell viability compromised, it is difficult to scale-up the application via this mode of transfection^19–21^. Chemical-based transfections, the typical alternative to viral vectors and electroporation, have come up short in transfecting primary cells, including natural killer (NK) and T cells. Lipofection, for example, has demonstrated extremely low efficiency as T cells demonstrate resistance to the standard chemical reagents^22,23^.

Mechanoporation, the delivery of cargo through transient pores created on the cell membrane by mechanical forces, offers a new avenue that can overcome the weaknesses of current transfection methods. Examples of such mechanical forces are shear stress, puncturing or compressions applied to the cell membrane, which can be generated by manipulating cells through microfluidic circuits or onto microneedles^19^. A first example of a mechanoporation method employed in gene therapy is microinjection, a technique where payload is precisely delivered into cells with high accuracy with micropipettes or nanoneedles. Microinjection, although capable of high transfection efficiency, requires accurate application of the cells onto a microneedle array, is highly cytotoxic, and faces challenges addressing high throughput^19^. Another rising mechanoporation method relies on individually squeezing cells with the help of microfluidic channels^24^. This technique allows cells to flow through constrictive microscopic channels smaller than the cell diameter and uses pressure-controlled gradient-driven flow to cause transient pores to deliver payload with high cell viability^19^. Many other microfluidics designs have emerged in the recent years including, microfluidic chambers utilizing the squeezing technique to create mechanical sheering combined with electrical field, microfluidic cell hydroporation, microfluidic vortex shedding and nanostraws^25–28^.

In the present work, we show a novel microfluidic device capable of efficiently delivering mRNA cargo into multiple human primary cell types without adverse effects using microfluidic mechanotransfection. The device uses a newly discovered cell behavior of volume exchange for convective transfection (VECT), an active transport phenomenon generated in response to large magnitude deformations at fast timescales in a manner that do not damage the cells^29,30^. In VECT, transient cell membrane pores and active transport of payload into the cells are created by flowing cells through microfluidic channels with a series of abrupt, rectangular cross-section ridges^29^. VECT has already proven useful in delivering large magnetic particles (i.e. diameter of around 40 nm) into cells for in vivo imaging^31^. We believe this technology can be straightforwardly scaled to patient scale by increasing the size and quantity of transfection elements without any alterations needed for the micromechanical environment.

## Results

### Gap size of ridge structures is a key variable to enable mRNA delivery via ultra-fast deformation in VECT device

**Figure 1:**
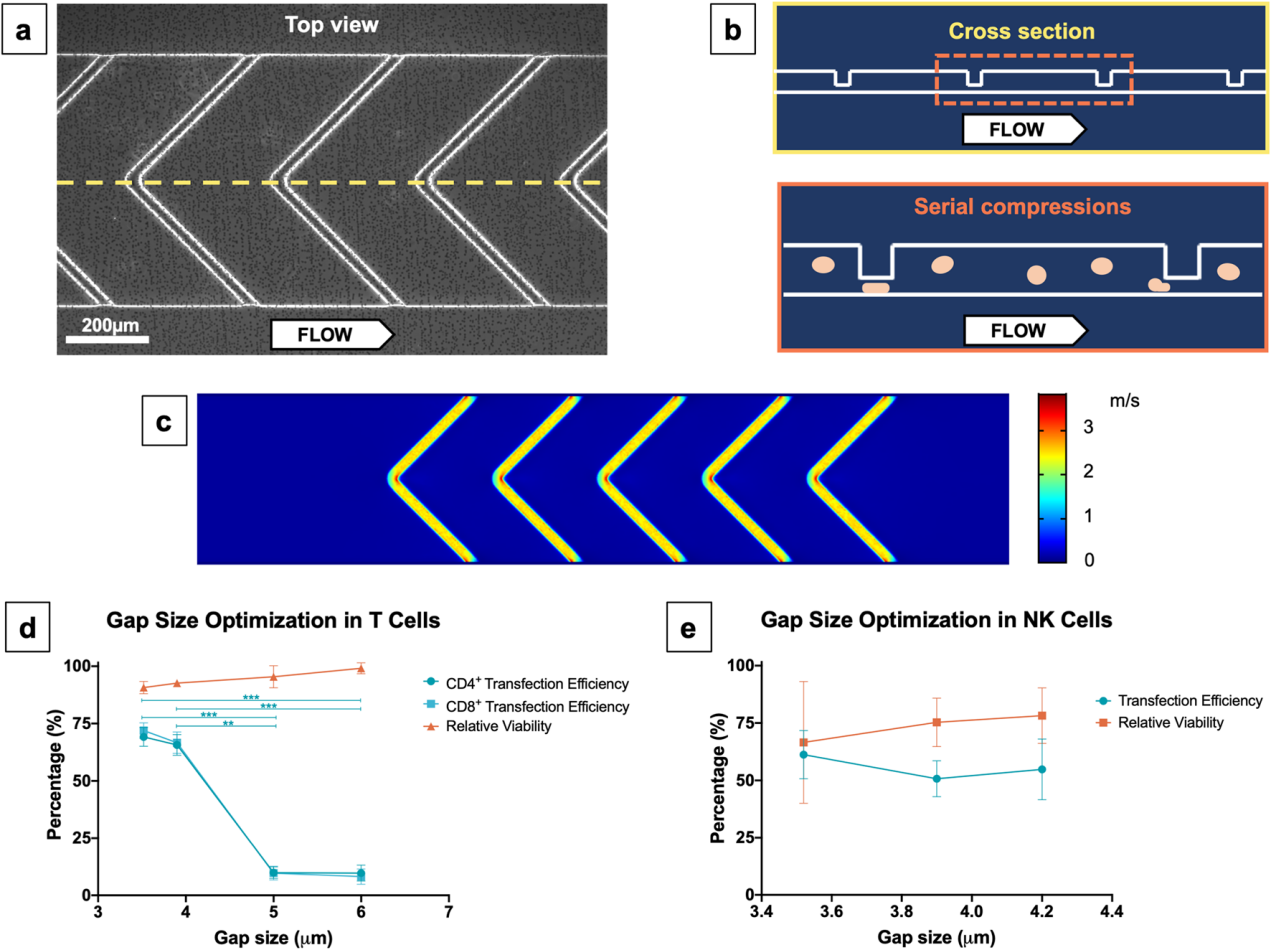
Optimization of a microfluidic device for volume exchange for convective transfection (VECT) in human primary cells. (**a**) Top-view microscopic image of the device, showing the shape of the ridge elements across the channel. (**b**) Top, representation of the cross section highlighted in yellow in (**a**), depicting how the ridges are distributed along the channel. Bottom, representation of the mechanical processing experienced by the cells. (**c**) Fluidic simulation of the velocity of the liquid being run in a working device. (**d**) Gap size optimization for the transfection of GFP-encoding mRNA in T cells performed with devices ranging from 3.5 to 6 µm (Mean ± SD, n = 3). Statistical analysis for the transfection efficiency in CD4^+^ and CD8^+^ T cells determined a significant difference between conditions (Welch ANOVA, P < 0.0001 for both cell types); Dunnett's T3 multiple comparisons tests between all conditions were performed, and differences found statistically significant are represented in the graph. No significant differences were found in the relative viability of the cells across the range of gap sizes tested (Welch ANOVA test, P = 0.0703). (**e**) Gap size optimization for the transfection of GFP-encoding mRNA in expanded NK cells performed with devices at 3.5, 3.9 and 4.2 µm gap size (Mean ± SD, n = 5–6), differences in transfection efficiency and relative viability were not found to be statistically significant according to Welch ANOVA test (P = 0.2481 and P = 0.6618, respectively).

We designed and manufactured a device capable of delivering mRNA payload into primary human cells by means of volume exchange for convective transfection (VECT), building on a microfluidic device reported previously^29,30^. In this new device, a microfluidic channel containing five chevron-shaped ridges (Fig. 1a,b) was able to abruptly compress cells at a short timescale (< 1 ms) when a suspension containing cells and payload in native media was flowed through the channel. When simulated in COMSOL, we predicted a laminar flow pattern behavior within the device, characterized by sudden, sharp changes in flow velocity at each ridge constriction (Fig. 1c). In order to achieve high mRNA transfection efficiencies while preserving cell viability, we optimized variables to include flow rate and height of the space under the ridges (i.e., “gap size”). Example of gap size optimization for the transfection of T cells and NK cells are provided in Fig. 1d (Supplementary Fig. 1) and Fig. 1e (Supplementary Fig. 2), respectively.

### VECT device for primary cells operates across a wide range of cell and payload concentrations

**Figure 2:**
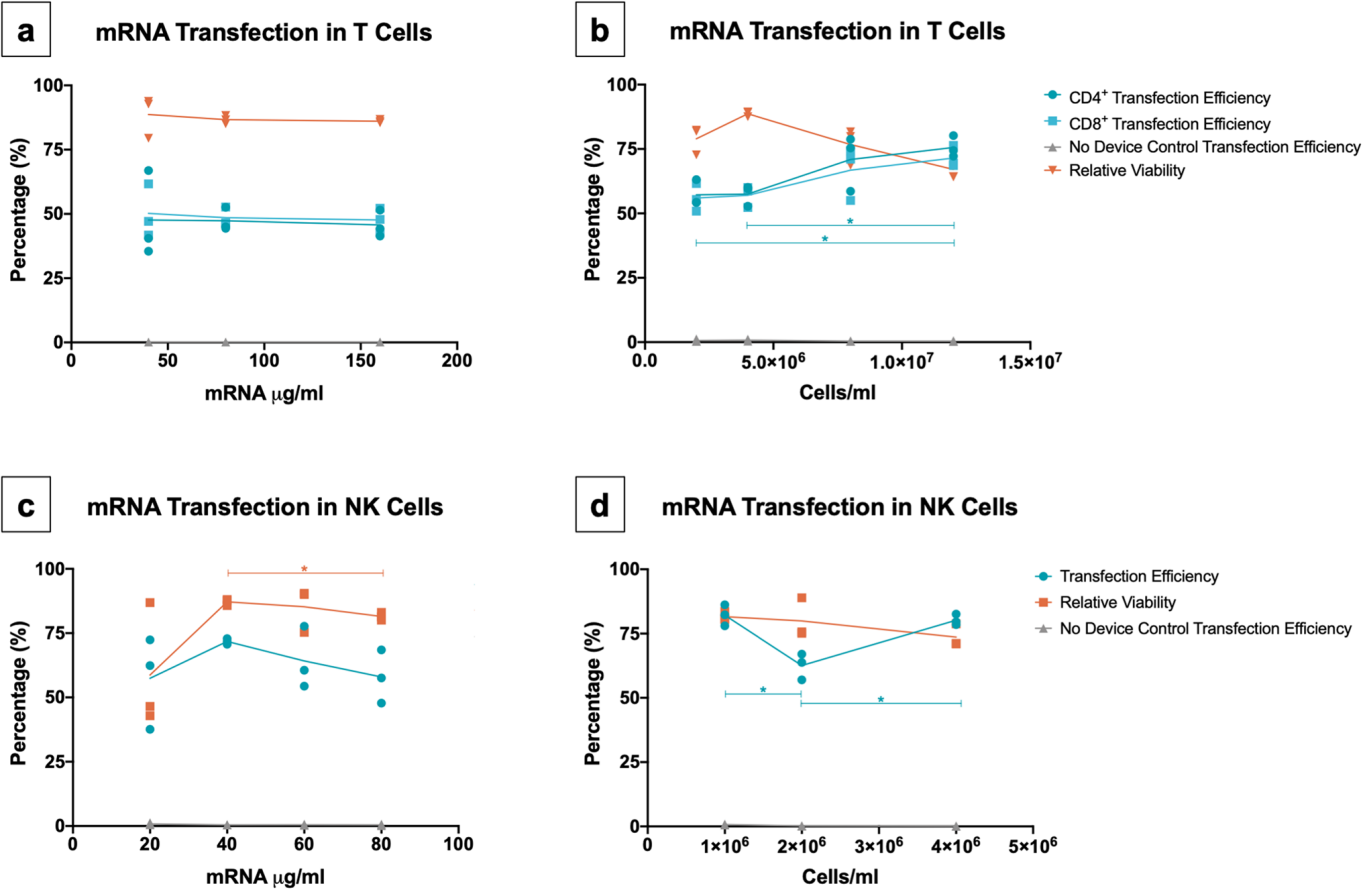
Operational ranges for the transfection of T cells and NK cells with mRNA employing the VECT microfluidic device. (**a**) Effect of mRNA concentration on mRNA VECT transfection of T cells (Mean and individual values, n = 3). Significant differences were not found in CD4^+^ transfection efficiency, CD8^+^ transfection efficiency, nor relative viability, across the range of mRNA concentrations used (Welch ANOVA test; P = 0.9319, P = 0.9305 and P = 0.7819, respectively). (**b**) Effect of cell concentration on mRNA VECT transfection of T cells (Mean and individual values, n = 3). Significant differences between conditions were found in CD4^+^ transfection efficiency and relative viability (Welch ANOVA test; P = 0.0226 and P = 0.0139, respectively). For CD4^+^ transfection efficiency and relative viability, Dunnett's T3 multiple comparisons test was performed across all concentrations tested, only significant differences are represented in the graph. No statistically significant differences were found in CD8^+^ transfection efficiency across the range of cell concentrations used (Welch ANOVA test; P = 0.0513). (**c**) Effect of mRNA concentration on mRNA VECT transfection of NK cells (Mean and individual values, n = 3). Significant differences were found in relative viability (Welch ANOVA test, P = 0.435), but not transfection efficiency (Welch ANOVA test, P = 0.2793), across the range of mRNA concentrations used. For relative viability, Dunnett's T3 multiple comparisons test was performed across all concentrations tested, and only significant differences are represented in the graph. (**d**) Effect of cell concentration on mRNA VECT transfection of NK cells (Mean and individual values, n = 3). Significant differences between conditions were found in transfection efficiency (Welch ANOVA test; P = 0.0215), but not in relative viability (Welch ANOVA test; P = 0.1635). In the case of NK transfection efficiency, Dunnett's T3 multiple comparisons test was performed across all concentrations tested, and significant differences are represented in the graph accordingly.

Having narrowed down the gap sizes necessary for the VECT microfluidic device to manufacture transfected cells at high yield, we decided to focus on understanding the operational ranges of the VECT microfluidic device in terms of payload and cell concentration in both T cells and NK cells. Potentially reducing the amount of mRNA required for transfection, as well increasing the operating cell concentration that is flowed inside the device, are complementary approaches to maximize the cost-efficiency of the transfection process. For T cells, transfection efficiency was sustained when reducing mRNA concentration from 160 to 40 µg/ml; while viability seemed to suffer slightly at higher concentrations of mRNA, this difference was not found to be statistically significant (Fig. 2a). Increasing operating T cell concentration from 2 × 10^6^ to 1.2 × 10^7^ cells/ml resulted in high transfection efficiencies for both CD4^+^ and CD8^+^ cells of at least 60%, when using a constant mRNA concentration of 70 µg/ml (Fig. 2b). Our results indicated that CD4^+^ transfection was significantly improved by the increase in cell numbers between the lower range of cell concentrations tested (2 × 10^6^ cells/ml and 4 × 10^6^ cells/ml), and the maximum concentration tested of 1.2 × 10^7^ cells/ml. There was a statistically significant difference in the relative viability values obtained at different cell concentrations, but Dunnett's T3 multiple comparisons test could not ascertain any given pair of relative viability values to be significantly different. In the case of NK cells, the effect of mRNA concentration on VECT was explored by employing mRNA ranging from 80 to 20 µg/ml (Fig. 2c). Results suggested a trend for transfection efficiency and cell viability peaking at 40 µg/ml of mRNA, however only relative viability was found to be significantly different at this mRNA concentration. Changes in NK cell concentration, tested between 1 × 10^6^ and 4 × 10^6^ cells/ml, only showed significant differences in transfection efficiency, with the lowest transfection (~ 60%) obtained at 2 × 10^6^ cells/ml, when using a constant mRNA concentration of 60 µg/ml (Fig. 2d). Further studies are required for both NK and T cell applications to understand the necessary minimum mRNA concentration for successful transfection, as well as the maximum processing capacity of the system in terms of cell concentration.

### Ultra-fast physical deformations do not impede normal T cell proliferation and T cell function

Preservation of cell functionality upon transfection is crucial for the potency of ex vivo gene therapies, such as T cell-derived products. In order to understand how VECT could affect normal T cell functionality, we decided to study T cell function upon microfluidic transfection by comparing the expansion, cytokine release, exhaustion and differentiation profiles of cells transfected with VECT to those of cells that do not experience compression (i.e. negative control). Maintenance of normal cell expansion upon gene modification is key to ensure the manufacturability of products requiring high cell numbers, such as CAR T cell products^32^. Functional cytokine expression plays a crucial role in concerting the T cell response together with innate and adaptive immune cell types necessary to clear pathogen infection^33^. T cell differentiation status has been described to play a key role in the outcome of T cell-based immunotherapy, with effector memory T cells (Tem) showing lesser persistence and antitumor immunity compared to stem cell memory T cells (Tscm) and central memory T cells (Tcm)^34^. Finally, T cell products that show higher T cell exhaustion at the time of infusion have been correlated with a poorer response against tumors^35^. We sought to understand the impact of VECT on these key aspects of T cell functionality.

**Figure 3:**
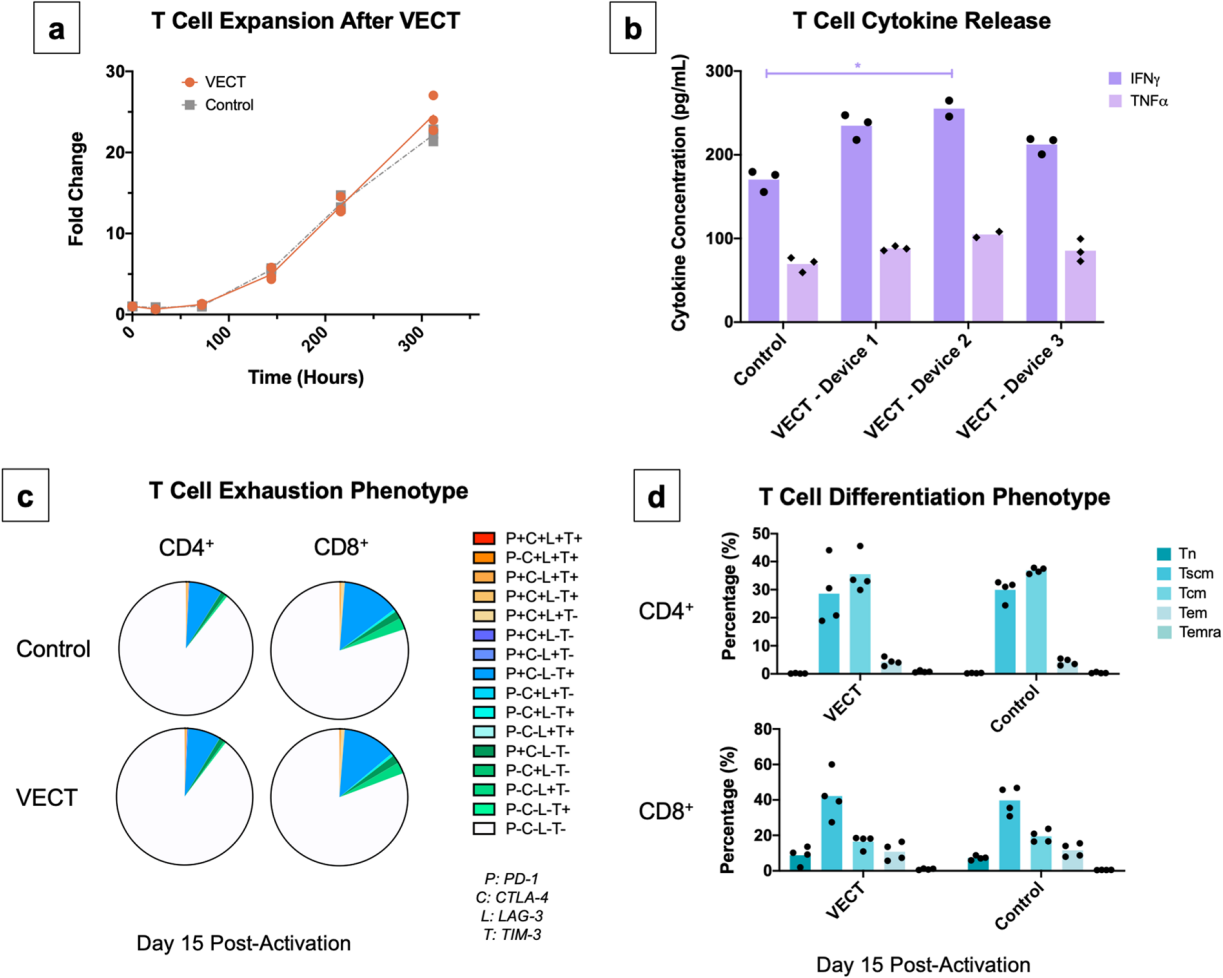
Cell functionality is preserved upon VECT. (**a**) Fold change in T cell expansion after VECT transfection closely followed the expansion seen in negative control cells (n = 3). (**b**) IFNγ and TNFα secretion was measured in cells re-activated 24 h after transfection, and results showed comparable expression between supernatant collected from a control cell sample and supernatants collected from three VECT-transfected samples (n = 3). Statistical analysis for IFNγ and TNFα secretion showed one significant difference in secretion levels of IFNγ between control cells and device 2 cell sample (Kruskal–Wallis test, p = 0.0036; Dunn's multiple comparisons test, p = 0.0247); (**c**) exhaustion profiles in expanded CD4^+^ and CD8^+^ T cell populations of both VECT and negative control cells, as measured by the detection of four exhaustion markers (PD-1, CTLA-4, LAG-3 and TIM-3) (n = 3). Results did not show evidence of exacerbated exhaustion after undergoing VECT, compared to the negative control. (**d**) T cell differentiation phenotype distribution was comparable between VECT and control cells for both CD4^+^ and CD8^+^ cells (n = 4). T cell subsets were measured as follows: Naïve (Tn: CD45RA^+^, CD45RO^−^, CD62L^+^, CD95^−^), Stem Cell Memory (Tscm: CD45RA^+−^, CD45RO^−^, CD62L^+^, CD95^+^), Central Memory (Tcm: CD45RA^−^, CD45RO^+^, CD62L^+^, CD95^+^), Effector Memory (Tem: CD45RA^−^, CD45RO^+^, CD62L^−^, CD95^+^), Effector Memory CD45RA^+^ (Temra: CD45RA^+^, CD45RO^−^, CD62L^−^, CD95^+^). T cells that could not be assigned to specific differentiation subsets were excluded from this graph.

First, VECT was employed to deliver GFP-encoding mRNA into T cells as described previously (data not shown). T cell functionality was then interrogated in these samples, with our findings indicating that T cell function was mostly preserved in primary T cells upon VECT transfection. No differences in fold expansion were observed between VECT and negative control cells (Fig. 3a, Supplementary Table 1), reaching a ~ 25-fold increase in cell numbers over a period of 15 days post-activation, indicating cells were ready to proliferate normally upon processing with the microfluidic device. When transfected cells were re-activated 24 h after VECT and cytokine expression was measured in a 48-h window, only IFNγ and TNFα secretion could be detected in the supernatant, and no significant differences were observable for these cytokines between supernatants collected from control cells and supernatants from VECT devices, except for significantly higher IFNγ secretion detected in one out of three VECT devices (Fig. 3b). Cell exhaustion profiles at the end of the 14-day expansion process were studied by detecting co-expression of PD-1, CTLA-4, LAG-3 and TIM-3 exhaustion markers (Fig. 3c). Results were comparable between VECT and negative control cells in both CD4^+^ and CD8^+^ compartments, demonstrating VECT does not stress T cells enough to precipitate exhaustion. Finally, when T cell differentiation was studied, both CD4^+^ and CD8^+^ T cells also provided a similar distribution of T cell differentiation immunophenotypes at 15 days after activation, regardless of whether T cells were transfected by VECT or did not experience compression in the negative control samples (Fig. 3d).

### VECT device enables transfection and co-transfection of mRNA molecules into human primary lymphocytes and hematopoietic stem cells at high efficiency

**Figure 4:**
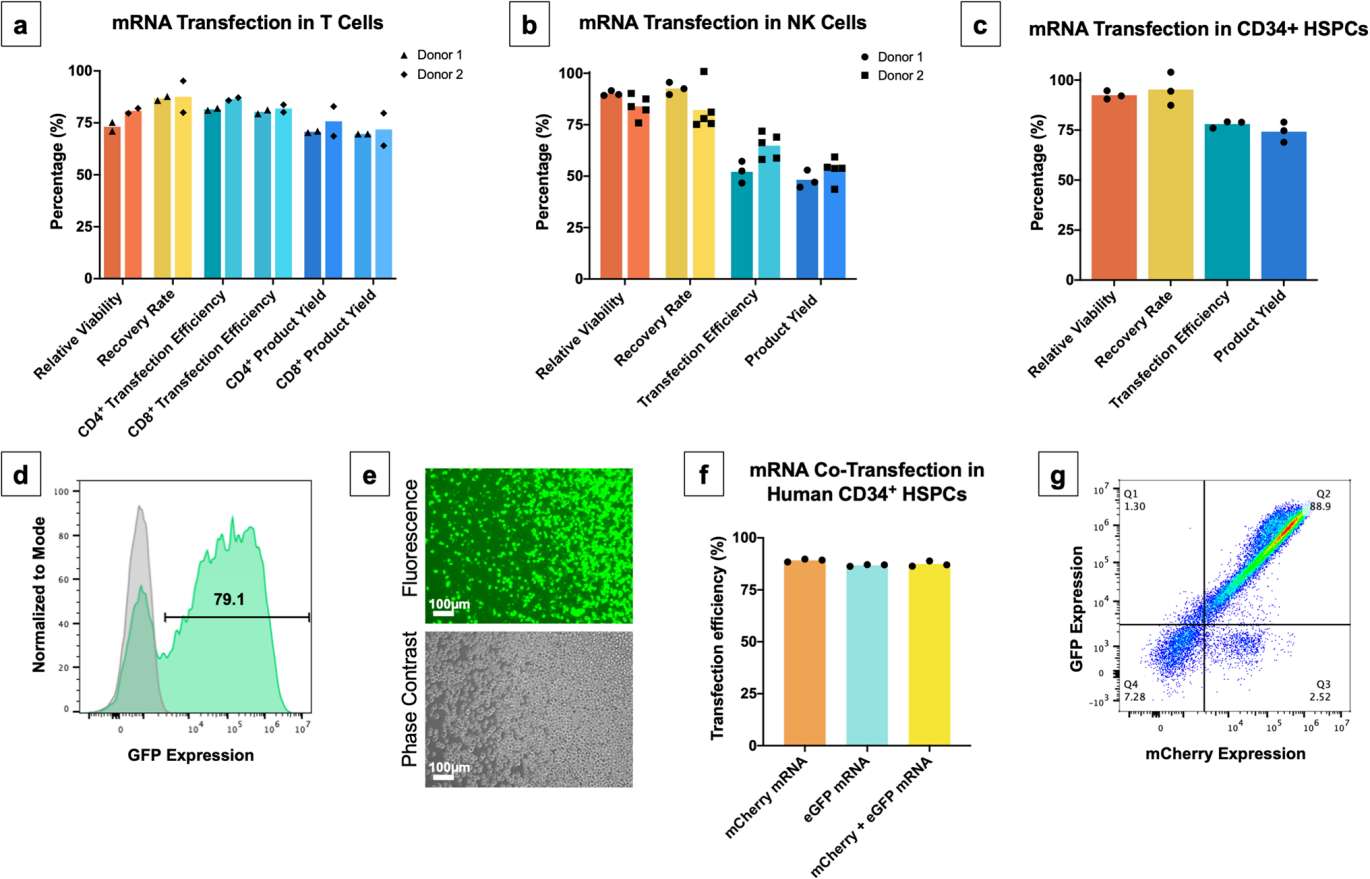
Optimized mRNA transfection results for T cells, NK cells and HSPCs; assessed by relative viability, recovery rate, transfection efficiency and product yield. (**a**) GFP mRNA transfection result in human T cells (two donors). T cells were isolated, activated and transfected with VECT. Transfection efficiency in CD4^+^ and CD8^+^ populations was obtained by flow cytometry after staining CD4^+^ and CD8^+^ markers with fluorescently labelled antibodies. (**b**) GFP mRNA transfection results for expanded human NK cells (two donors). (**c**) GFP mRNA transfection results for mobilized peripheral blood CD34^+^-selected human HSPCs (one donor). (**d**) Representative histogram of HSPC transfection with GFP mRNA, where transfection efficiency was measured by means of detection of GFP fluorescence by flow cytometry. (**e**) Representative picture of HSPC GFP fluorescence signal as captured under fluorescence microscope. (**f**) Comparison of the delivery of single mRNA molecules encoding for mCherry and eGFP versus co-delivery of the same two mRNA molecules in HSPCs. (**g**) Representative flow cytometry dot plot for the co-delivery of eGFP mRNA (y axis) and mCherry mRNA (x axis).

Our current best performing conditions for T cell and NK cell transfection using VECT are shown in Fig. 4, together with the translation of mRNA VECT into mobilized peripheral blood CD34^+^ HSPCs. The performance of the transfection process was characterized by measuring relative viability, recovery rate, transfection efficiency and product yield in these three cell types of therapeutic interest. Our findings indicated that optimal conditions for VECT transfection of mRNA were somewhat different between T cells, NK cells, and mobilized peripheral blood CD34^+^ hematopoietic stem and progenitor cells (HSPCs), possibly reflecting key differences in the biomechanical properties of these cell types. Data demonstrating successful transfection of mRNA into primary T cells (Fig. 4a) and NK cells (Fig. 4b) were generated across two independent donors for each cell type. In the case of T cells, VECT resulted in high relative viabilities and recovery rates (~ 75%), with mRNA transfection efficiency reaching about 80%, and product yields of approximately 70% in both CD4^+^ and CD8^+^ T cells. For NK cells, VECT resulted in higher relative viability and recovery rates (> 80%) but lower transfection efficiency (~ 60%) and product yield (~ 50%) than T cells.

When performing VECT on HSPCs (Fig. 4c), we observed the microfluidic device was capable of retaining high relative viability and recovery rate (> 90%), while achieving high transfection efficiency (~ 75%), and total product yield (> 70%). Transfection efficiency in HSPCs was measured by the appearance of GFP-positive cells under flow cytometry analysis (Fig. 4d) and confirmed visually under a fluorescence microscope (Fig. 4e). Some therapeutic applications of mRNA transfection, such as gene editing with ZFN or TALEN, require simultaneous co-expression of two different proteins in a target cell. In order to demonstrate the capacity of the device to co-deliver two independent mRNA molecules, we decided to measure the transfection efficiency of two different mRNA molecules coding for mCherry and eGFP protein, respectively. Transfection efficiencies for mCherry mRNA alone, eGFP mRNA alone, and for the co-delivery of both mRNA molecules were high (> 85%) and consistent across all three conditions (Fig. 4f). In fact, when analyzed by flow cytometry, co-transfection showed a tight linear correlation between mCherry and eGFP expression, except for a small population (< 3% of the total cells) showing single positive signal for mCherry expression (Fig. 4g).

## Discussion

Transfection of mRNA offers a simpler, less genotoxic and more rapid platform for expression of transgenes compared to predominant DNA-based methods^12,36,37^. Ex vivo delivery of mRNA into human primary blood cells is currently enabled by electroporation technologies, which have raised concerns around product scalability and translation into the clinics due to toxicity and low cell viability^19–21^. In the present work, we have demonstrated the capacity of a novel microfluidic device to successfully transfect mRNA into human primary T cells, NK cells, and CD34^+^ HSPCs by means of volume exchange for convective transfection (VECT). According to our simulations, the device operated under a laminar flow regime that resulted in sharp, short-lived changes in cell velocity upon encountering ridges that constrict the channel height under the ridge (i.e. gap size) to subcellular dimensions. We found the gap size was a critical factor during optimization of the delivery of mRNA into primary cells. However, our results also pointed towards a level of tolerance in the gap size required for transfection, possibly facilitating the consistent device performance observed across donors. The role of cell biomechanics in designing optimal device designs remains to be explored in primary cells.

Having demonstrated the importance of gap size in the VECT device to achieve delivery of mRNA, we sought to explore the operational capacity of the device. Variable ranges for both payload concentration and cell concentration were explored in VECT transfection. The desired number of T cells or NK cells were placed in their respective native media, together with the required amount of mRNA, and processed through the microfluidic device. We determined we could readily modify the number of cells and the amount of payload employed in the transfection. To assess the operational capacity of the VECT device in terms of cell concentration, we explored a range spanning an order of magnitude above typical cell culture densities (e.g. T cell seeding at 1 million cells/ml^38^), and our results suggested that the process can be implemented in current cell culture workflows without the need to concentrate or dilute cells. When payload concentration was interrogated, we could see successful transfection of mRNA with as low as 40 μg/mL of mRNA, comparable or below the range of concentrations reported to work in the context of electroporation^39,40^.

T cell function upon VECT was interrogated next, and successful processing of T cells did not show a detrimental impact on T cell function in terms of cell expansion and exhaustion. We believe these results strongly support the future use of the technology for rapid generation of therapeutic T cell products such as mRNA CAR T cells. Currently, commercially approved CAR T cell products rely on integrating viral vectors able to insert a CAR transgene into the genome of the cells^41,42^. However, there is a growing body of knowledge supporting the use mRNA expression for the rapid manufacture of CAR T cells. With mRNA CAR T cells, transient CAR expression is exploited to achieve time-constrained anti-tumor activity, addressing safety concerns related to on-target off-tumor toxicity, and long-term expression of the CAR in patients^43^. The same is also true for other CAR-based cell products under development, such as mRNA CAR NK cells^44^.

We found optimized VECT conditions to be able to manufacture mRNA-transfected T cell and NK cell products with high productivity. Approximately 70% of T cells and 50% of NK cells put into the device were transfected and recovered successfully from the system. Moreover, learnings from T cells and NK cells allowed us to readily translate mRNA VECT into CD34^+^ HSPCs, where productivity was 70%, close to that observed in T cells. Taken together, the results obtained in three different primary cell types of clinical interest suggest VECT technology is straightforward to translate and tune to different human cell types. We believe developing our work with these and other cell types could result in the ability to more rapidly translate (or even predict) optimal VECT conditions in novel cell types.

Co-delivery of two different mRNA payloads was tested in our HSPCs process and resulted in a transfection efficiency equivalent to that observed in single transfections of either mRNA, with most cells achieving co-expression in a linearly correlated manner. Transient expression of mRNA can be exploited to permanently modify the genome for mRNA payload encoding gene editing molecules such as zinc finger nucleases, transcription activator-like effector nucleases (TALEN) or CRISPR/Cas9 systems^17^. Indeed, transient ‘hit-and-run’ expression of such molecules is desirable to reduce risks of off-target mutagenesis and possible immune responses against gene editing proteins^20,45,46^. An interesting parallel to this application can be found with the use of ‘hit-and-run’ mRNA expression to achieve inheritable modifications of the epigenome, employing mostly variants of the same gene editing proteins^47^ (e.g. CRISPR/Cas9). For conventional ZFN, TALEN and CRISPR/Cas9 gene editing approaches, the presence of two distinct molecules is required at any given target site: either a combination of two ZFN proteins, two TALEN proteins, or delivery of a Cas9 protein together with a guide RNA molecule^17^. We have demonstrated here the potential to address such gene editing applications by successfully co-delivering two different molecules of mRNA at the same time. We are aware that more advanced gene editing approaches are seen in the clinics requiring co-delivery of a higher number of payloads targeting multiple loci^48^, and further studies are ongoing to understand delivery limitations for the device when transfecting more than two payloads at once.

We are aware of multiple platforms pursuing transfection via mechanoporation relying on mechanisms of delivery different than VECT. Individually squeezing cells with the help of microfluidic channels has not proven to achieve efficient delivery of nucleic acids (e.g. mRNA), unless mechanical and electrical stress are applied concomitantly in a single transfection process^24,25^. Microinjection platforms require manipulation of single cells with high precision in a microfluidic array of needles and are therefore hard to scale because they are limited by the total surface of the device^19^. We understand microfluidic vortex shedding to be the platform that most closely matches the capabilities of VECT, reporting transfection efficiencies of up to 63% in human T cells when using high concentration of mRNA (160 µg/ml)^27^. We have provided here evidence of superior mRNA transfection in multiple human primary cells, with up to 80% transfection efficiency in the case of T cells, while employing significantly lower amounts of mRNA payload (60–70 µg/ml). To the best of our knowledge, the current work represents the most efficient mRNA mechanoporation platform in human primary cells reported in literature up to date, both in terms of transfection efficiency, high cell number processing capability, and payload concentration requirements.

The present work shows the VECT microfluidic device can be employed in a wide range of ex vivo mRNA-based applications, cell carriers, and operating conditions. We and others have previously demonstrated the capacity of the VECT device to increase its throughput by multiplexing the number of channels employed simultaneously^29–31^. Because the microfluidic element is not changed but parallelized, we expect multiplexing of the same VECT channel to scale our results directly from low to high throughput. We next aim to demonstrate the scalability of the present device to achieve transfection at clinical scale; namely, capacity to manufacture hundreds of millions of mRNA-transfected T cells and NK cells with the same high productivity as described in the present work.

## Methods

### Device manufacturing

For the optimization studies, microfluidic devices were made with polydimethylsiloxane (PDMS) replica molding using SU-8 photoresist patterned onto a silicon substrate. PDMS was mixed at a 10:1 ratio of Sylgard 184 silicone elastomer with curing agent, degassed and poured onto the mold and cured at 80 °C for 2 h. Outlet holes were punched using a biopsy punch and the chip bonded to a glass slide after an air plasma treatment. When explicitly stated in the results, once the optimal device design was determined, microchannels were fabricated in a silicon wafer through a two-step deep reactive ion etching process, which was then bonded to a Pyrex wafer via anodic bonding. Inlets and outlet ports on the Pyrex wafer were created by sand blasting and each device was cut via a precision dicing saw.

### T cell isolation

PBMCs (purchased from AllCells) were magnetically separated with Human Pan T Isolation Kit (Miltenyi Biotec). The newly isolated T cells were then cultured in complete T cell medium, composed of TexMACS (Miltenyi Biotec) supplemented with IL-2 (PeproTech, 100 IU/ml) and Penicillin Streptomycin (1:100). Cells were activated with human T cell TransACT (Miltenyi Biotec) for approximately 20 h before transfection.

### T cell VECT transfection

Activated T cells were flown through a microfluidic device at a flow rate of 400 µl/min. Microfluidic devices were first passivated with TexMACS (Miltenyi Biotec). T cells were spun down at 300G for 5 min and washed with PBS. Cells were resuspended between 2 and 3 million cells/ml in T cell VECT Buffer: TexMACS (Miltenyi Biotec), and 1:1,000 Superase RNase inhibitor (Invitrogen). Before VECT transfection, CleanCap eGFP mRNA (TriLink) payload was added to the cells. 250 µl of reaction were processed per channel and plated in a 24-well plate already containing 250 µl complete T cell medium. Unless stated otherwise, the mix of T cells and payload was flowed through a microfluidic device of 4 µm gap size device at a flow rate of 800 µl/min, and an mRNA concentration of 70 µg/ml. Cell counts were performed after VECT transfection with NucleoCounter NC-3000 (ChemoMetec), using A8 slides (ChemoMetec) and solution 13 (ChemoMetec) as per manufacturer instructions.

### T cell flow cytometry

Transfection efficiency readouts were performed with flow cytometry 18–24 h post experiment. FACS buffer was prepared by adding fetal bovine serum (2%) into DPBS buffer. Sample and negative control (no device, ND) cells were plated into a 96-well V-bottom plate and spun down at 300G for 5 min. The wells were then stained with FACS buffer containing Miltenyi Biotec REAfinity human antibodies for the detection of CD4 (VioBlue; 1:50) and CD8 (APC; 1:100), for 10 min at 4 °C in the dark. The plates were washed twice with FACS buffer before 100 μl SYTOX AADvanced dead cell staining solution (1:1000 SYTOX AADvanced diluted in FACS Buffer) were added and analyzed immediately with CytoFLEX S flow cytometer (Beckman Coulter).

In order to measure T cell cytokine release, samples were from cells transfected with VECT or negative control cells were centrifuged for 5 min at 300*g*, and supernatant from each sample was collected and directly stored at − 80 °C until ready to be measured. Cytokines in these samples were quantified by flow cytometry by using the Human Th1/Th2 Cytokine Cytometric Bead Array (CBA) Kit II (BD Biosciences), as per manufacturer’s instructions.

T cell exhaustion was measured on day 14 post-VECT transfection. Sample, negative control (no device, ND), fluorescence-minus-one (FMO), isotype, and SYTOX AADvanced-single stain cells were plated into two 96-well V-bottom plate and spun down (300G for 5 min). Sample and ND cells were stained with FACS buffer containing Miltenyi Biotec REAfinity human antibodies for the detection of LAG3 (VioBlue; 1:50), CD8 (VioGreen; 1:50), CTLA4 (PE; 1:50), PD1 (PE-Vio770; 1:50), TIM3 (APC; 1:50), and CD4 (APC-Vio770; 1:800). 8 fluorescence minus one (FMO) wells were stained with the same Miltenyi Biotec REAfinity human antibodies. The one isotype well was stained with Mintenyi Biotec REA Control Antibodies of the same fluorophores at the same dilutions. The plate was incubated in the dark in a 4 °C fridge for 10 min. Sample wells were washed with FACS buffer three times before being resuspended in 100 μl SYTOX AADvanced dead cell staining solution (1:1000 SYTOX AADvanced diluted in FACS Buffer). A compensation panel consisting of seven wells was added onto the plate, with 50 µl of Miltenyi Biotec anti-REA compensation beads (1:1, negative and positive beads) in each well. One single stain was added into each compensation well, employing the same REAfinity human antibodies used in the samples. The plate containing cell samples and compensation beads was incubated in the dark in a 4 °C fridge for 10 min. 100 µl of FACS buffer were added to the compensation bead wells, before analyzing results with CytoFLEX S flow cytometer (Beckman Coulter). T cell differentation was similarly measured on day 14 post-VECT transfection, but employing Miltenyi Biotec REAfinity human antibodies for the detection of CD4 (VioBlue; 1:50), CD45RO (VioGreen; 1:50), CD62L (PE; 1:50), CD45RA (PE-Vio770; 1:200), CD8 (APC; 1:100), and CD95 (APC-Vio770; 1:100).

### NK cell isolation and expansion

PBMCs were supplied commercially (AllCells) and magnetically separated with NK Cell Isolation Kit (Miltenyi Biotec). Upon isolation, NK cells were cultured in complete NK medium, prepared with NK MACS media (Miltenyi Biotec) supplemented with human serum (1:20), penicillin streptomycin (1:100), NK supplement (1:100, Miltenyi Biotec), and IL-2 (500 IU/ml). Cells were transferred into a 24-well G-REX (Wilson Wolf) five days after isolation, and fresh NK media was then added every 2–3 days to ensure cell health. Purity was measured by flow cytometry using Miltenyi Biotec’s CD56 REAfinity (PE-Vio770; 1:50) at day nine of the NK cell culture. Cells were used for transfection 15–18 days after isolation.

### NK cell VECT transfection

Before transfection, devices were passivated with NK MACS medium. NK cells were washed with DPBS, and prepared in NK MACS medium with 1:1000 Superase RNase inhibitor (Invitrogen), at a concentration of 2 million cells/ml. CleanCap eGFP mRNA (TriLink) was added to the cells with a concentration ranging from 20 to 80 µg/ml. Unless stated otherwise, the mix of NK cells and payload was flowed through a microfluidic device of 3.5 µm gap size device at a flow rate of 800 µl/min, and an mRNA concentration of 60 µg/ml. 250 µl of sample were processed per channel, and plated in a 24-well plate already containing 250 µl of 2× complete NK medium. Cell counts were performed after VECT transfection with NucleoCounter NC-3000 (ChemoMetec), using A8 slides (ChemoMetec) and solution 13 (ChemoMetec) as per manufacturer instructions. Cells were placed in the incubator for cell culture.

### NK cell flow cytometry

On day nine of NK cell culture, the purity of the NK cells was measure by flow cytometry. FACS buffer was prepared by adding fetal bovine serum (2%) into DPBS buffer. Samples of the culture were plated into two wells in a 96-well V-bottom plate and spun down at 300G for 5 min. One well was then stained with FACS buffer containing antibodies for detection of CD56 (PE-Vio770; 1:50) for 10 min in the dark at 4 °C. The plates were washed twice with FACS buffer before 100 μl of FACS buffer was added and analyzed immediately with CytoFLEX S flow cytometer (Beckman Coulter).

Transfection efficiency was assessed the day (18–24 h) post experiment by flow cytometry. FACS buffer was prepared by adding fetal bovine serum (2%) into DPBS buffer. Sample and negative control (no device, ND) cells were plated into a 96-well V-bottom plate and spun down at 300G for 5 min. The plates were then washed twice with FACS buffer before 100 μl SYTOX AADvanced dead cell staining solution (1:1000 SYTOX AADvanced diluted in FACS Buffer) were added and processed through a CytoFLEX S flow cytometer (Beckman Coulter).

### Thawing of CD34^+^ HSPCs

Mobilized peripheral blood CD34-selected hematopoietic stem and progenitor cells (mPB CD34^+^ HSPCs, purchased from AllCells) were taken from liquid nitrogen freezer and placed into a 37 °C water bath to thaw. 10 ml of HSPC complete media were prepared by mixing 10 ml Lonza X-VIVO 10, 10 µl Stem Cell Factor (SCF; 100 µg/ml), 10 µl Thrombopoietin (TPO; 100 µg/ml), 10 µl FMS-like tyrosine kinase 3 ligands (Flt-3L; 100 µg/ml), 100 µl of GlutaMAX-I (100X), and 100 µl of PenStrep (10,000 U/ml). HSPC media was mixed well and filtered through a 0.2 µm syringe filter. 20 ml of HSPC washing medium were prepared by mixing 20 ml Lonza X-VIVO 10, 200 µl of GlutaMAX-1 (100×), and 100 µl of PenStrep (10,000 U/ml) per frozen aliquot cells. Once thawed, cells were resuspended gently into a 50 ml conical tube containing the 20 ml of HSPC washing medium at room temperature. Cells were spun at 300G for 7 min, the supernatant was decanted, and t HSPC complete medium was used to resuspend the cell pellet at a cell density of approximately 1 million cells/ml. Cells were incubated overnight at 37 °C, 5% CO_2_.

### CD34^+^ HSPC culture

24 h after thawing the HSPCs, cells were counted to ensure cell quality. Cells were returned to the incubator at 37 °C, and 5% CO_2_ for another 24 h.

### CD34^+^ HSPC VECT transfection

VECT medium (X-VIVO 10 Media (Lonza), and 1:1000 Superase RNase inhibitor (Invitrogen)) and complete HSPC medium (see Thawing of CD34^+^ HSPCs) were prepared. VECT microfluidic devices were passivated with 0.5 ml VECT medium per channel. HSPCs were spun at 300*g* for 5 min, washed with nuclease-free DPBS, and placed in HSPC VECT medium at 2 million cells/ml. Before VECT, CleanCap eGFP mRNA (TriLink) was added to the cells to achieve a final concentration of 160 µg/ml. For each sample, 200 µl HSPCs were flown through a 4 µm gap size device at a rate of 800 µl/min, into another 200 µl of pre-incubated 2× complete HSPC medium. Cell counts were performed after VECT transfection with NucleoCounter NC-3000 (ChemoMetec), using A8 slides (ChemoMetec) and solution 13 (ChemoMetec) as per manufacturer instructions. Lastly, samples were incubated at 37 °C, 5% CO_2_for 24 h before analysis.

### HSPC flow cytometry

Transfection efficiency was assessed the day after transfection (18–24 h post experiment) by flow cytometry. FACS buffer was prepared by adding fetal bovine serum (2%) into DPBS buffer. Samples, negative control (no device, ND), isotype, and unstained cells were plated into a 96-well V-bottom plate. Cells were spun down at 300G for 5 min. Sample, and ND cells was stained with FACS buffer containing Miltenyi REAfinity human antibodies for CD34 (VioBlue; 1:50) and CD38 (APC; 1:50) detection. Isotype control was incubated with Mintenyi Biotec REA control antibodies for VioBlue and APC fluorophores at the same equivalent dilutions. The plate was incubated in the dark at 4 °C for 10 min. Two more washes at 300G for 5 min were performed. 100 µl SYTOX AADvanced dead cell staining solution (1:1000 SYTOX AADvanced diluted in FACS buffer) were added to sample, ND, and isotype wells. 100 µl FACS buffer was added to the unstained well. The plate was then readout on a CytoFLEX S flow cytometer (Bechman Coulter).

### Formulae

Relative viability was calculated as:

$${\text{relative}}\;{\text{viability}}\left( \% \right) = {\text{Sample}}\;{\text{viability}}/{\text{No}}\;{\text{device}}\;{\text{viability}} \times 100$$

Recovery was calculated as:

$${\text{Recovery}}\;{\text{rate}}\left( \% \right) = {\text{Sample}}\;{\text{live}}\;{\text{cell}}\;{\text{concentration}}/{\text{No}}\;{\text{device}}\;{\text{live}}\;{\text{cell}}\;{\text{concentration}} \times 100$$

Product yield was calculated as:

$${\text{Product}}\;{\text{yield}}\left( \% \right) = {\text{Transfection}}\;{\text{efficiency}} \times {\text{Recovery}}\;{\text{rate}}$$

## Supplementary Information

Supplementary Information.
