# Supplementary material for: Microfluidic transfection of mRNA into human primary lymphocytes and hematopoietic stem and progenitor cells using ultra-fast physical deformations
Source: Sci Rep. 2021 Nov 1;11:21407. doi: 10.1038/s41598-021-00893-4 (PMC8560772; doi:10.1038/s41598-021-00893-4)
Supplement: Supplementary file 1 — Supplementary Information. [file 41598_2021_893_MOESM1_ESM.docx]

**Supplementary Data**

**Supplementary Table 1.** Values characterizing T cell expansion in cells that have experienced VECT transfection, and negative control cells. Triplicate values are provided for a total of six time points spanning a total of 312 hours. For each replicate, fold change expansion has been calculated by normalized all cell counts to their respective cell count value at the starting time point.

|  | **VECT** | | | | | | **Control** | | | | | |
| --- | --- | --- | --- | --- | --- | --- | --- | --- | --- | --- | --- | --- |
|  | **Replicate 1** | | **Replicate 2** | | **Replicate 3** | | **Replicate 1** | | **Replicate 2** | | **Replicate 3** | |
| **Time (h)** | **Cell Counts** | **Fold-Change** | **Cell Counts** | **Fold-Change** | **Cell Counts** | **Fold-Change** | **Cell Counts** | **Fold-Change** | **Cell Counts** | **Fold-Change** | **Cell Counts** | **Fold-Change** |
| 0 | 1.48E+06 | 1.00 | 1.46E+06 | 1.00 | 1.28E+06 | 1.00 | 1.82E+06 | 1.00 | 1.86E+06 | 1.00 | 1.97E+06 | 1.00 |
| 24 | 1.00E+06 | 0.68 | 9.22E+05 | 0.63 | 8.75E+05 | 0.68 | 1.27E+06 | 0.70 | 1.69E+06 | 0.91 | 1.77E+06 | 0.90 |
| 72 | 1.88E+06 | 1.27 | 1.69E+06 | 1.16 | 1.71E+06 | 1.34 | 2.08E+06 | 1.14 | 1.80E+06 | 0.97 | 2.14E+06 | 1.09 |
| 144 | 6.47E+06 | 4.37 | 6.79E+06 | 4.65 | 7.44E+06 | 5.81 | 9.68E+06 | 5.32 | 1.04E+07 | 5.59 | 1.13E+07 | 5.74 |
| 216 | 1.88E+07 | 12.70 | 1.89E+07 | 12.95 | 1.86E+07 | 14.53 | 2.37E+07 | 13.02 | 2.74E+07 | 14.73 | 2.61E+07 | 13.25 |
| 312 | 3.55E+07 | 23.99 | 3.32E+07 | 22.74 | 3.46E+07 | 27.03 | 4.06E+07 | 22.31 | 4.25E+07 | 22.85 | 4.21E+07 | 21.37 |


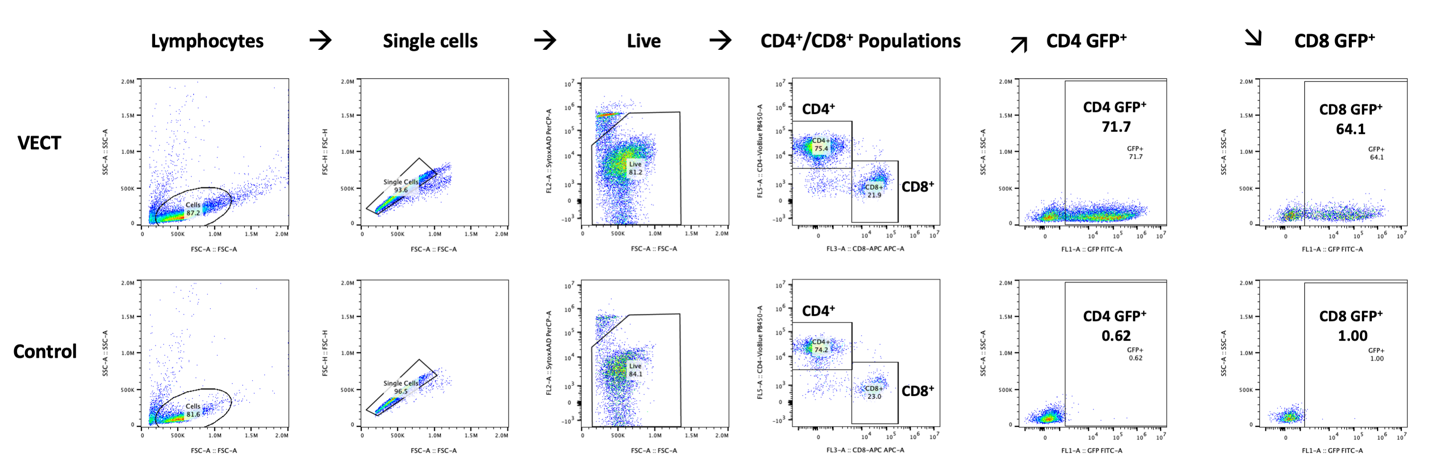


**Supplementary Figure 1.** Example of Flow Cytometry gating followed to quantify GFP^+^ CD4^+^ and CD8^+^ T cells. First, Lymphocyte populations were selected by size and granularity (FSC-A, SSC-A). From these selection, single cells were isolated (FSC-A, FSC-H). SytoxAADvanced was then employed to gate out dead cells (FSC-A, PerCP-A). CD4^+^ and CD8^+^ were discriminated by antibody staining (APC-A, PB450-A). Finally, each CD4^+^ and CD8^+^ populations were plotted as a GFP FITC-A histogram and the background signal in control CD4^+^ and CD8^+^ samples were employed to set up the GFP^+^ transfection efficiency gate in transfected CD4^+^ and CD8^+^ cells.


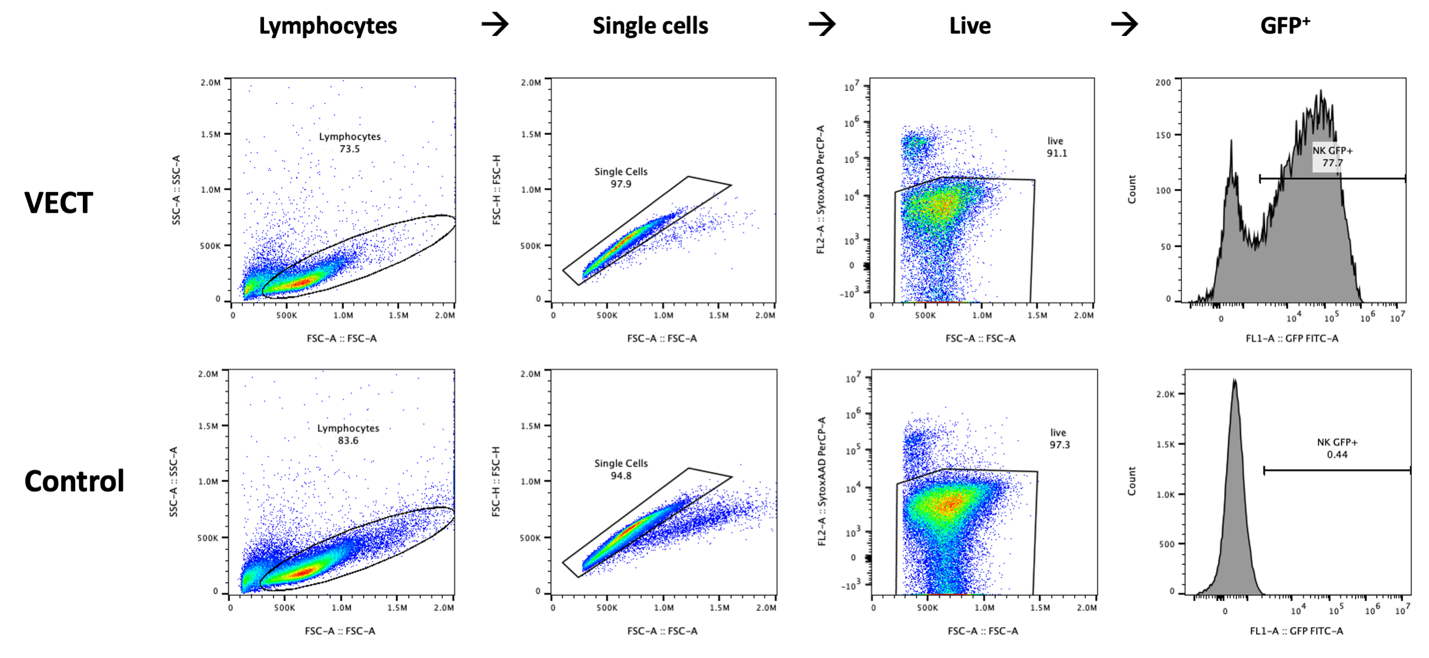


**Supplementary Figure 2.** Example of Flow Cytometry gating followed to quantify GFP^+^ NK cells. First, Lymphocyte populations were selected by size and granularity (FSC-A, SSC-A). From these selection, single cells were isolated (FSC-A, FSC-H). SytoxAADvanced was then employed to gate out dead cells (FSC-A, PerCP-A). Finally, the single cell alive population was plotted as a GFP FITC-A histogram and the background signal in control samples was employed to set up the GFP^+^ transfection efficiency gate.
